# Optimism/pessimism and health-related quality of life during pregnancy across three continents: a matched cohort study in China, Ghana, and the United States

**DOI:** 10.1186/1471-2393-9-39

**Published:** 2009-09-01

**Authors:** Cheryl A Moyer, Huixia Yang, Yao Kwawukume, Anu Gupta, YuChun Zhu, Isaac Koranteng, Yasmin Elsayed, YuMei Wei, Jonathan Greene, Cecilia Calhoun, Geraldine Ekpo, Megan Beems, Megan Ryan, Richard Adanu, Frank Anderson

**Affiliations:** 1 Global REACH, University of Michigan, 717 E. Huron, Suite 1E; Ann Arbor, MI 48104; 2 Department of Obstetrics and Gynecology, Peking University First Hospital, No.8 XiShiKu St. XiCheng District, Beijing, PR China, 100034; 3 Department of Obstetrics and Gynecology, University of Ghana, University of Ghana Medical School, PO Box GP4236, Accra, Ghana; 4 University of Michigan Medical School, 1500 E. Medical Center Drive. Ann Arbor, MI 48109; 5 Department of Obstetrics and Gynecology, University of Michigan Medical School, 1500 E. Medical Center Drive. Ann Arbor, MI 48109

## Abstract

**Background:**

Little is known about how optimism/pessimism and health-related quality of life compare across cultures.

**Methods:**

Three samples of pregnant women in their final trimester were recruited from China, Ghana, and the United States (U.S.). Participants completed a survey that included the Life Orientation Test - Revised (LOT-R, an optimism/pessimism measure), the Short Form 12 (SF-12, a quality of life measure), and questions addressing health and demographic factors. A three-country set was created for analysis by matching women on age, gestational age at enrollment, and number of previous pregnancies. Anovas with post-hoc pairwise comparisons were used to compare results across the cohorts. Multivariate regression analysis was used to create a model to identify those variables most strongly associated with optimism/pessimism.

**Results:**

LOT-R scores varied significantly across cultures in these samples, with Ghanaian pregnant women being the most optimistic and least pessimistic and Chinese pregnant women being the least optimistic overall and the least pessimistic in subscale analysis. Four key variables predicted approximately 20% of the variance in overall optimism scores: country of origin (p = .006), working for money (p = .05); level of education (p = .002), and ever being treated for emotional issues with medication (p < .001). Quality of life scores also varied by country in these samples, with the most pronounced difference occurring in the vitality measure. U.S. pregnant women reported far lower vitality scores than both Chinese and Ghanaian pregnant women in our sample.

**Conclusion:**

This research raises important questions regarding what it is about country of origin that so strongly influences optimism/pessimism among pregnant women. Further research is warranted exploring underlying conceptualization of optimism/pessimism and health related quality of life across countries.

## Background

The psychosocial constructs of optimism and pessimism have been under study for several decades. Optimism is associated with more active coping strategies, lower levels of psychological distress [1-5], health-enhancing behavior [6], higher immune functioning [7], better health outcomes[8] and even lower mortality. [9-11] On the other hand, pessimism has been shown to have prophylactic effects in certain circumstances. In particular, pessimism can insulate people from the psychological consequences of failure, including anxiety, depression, and diminished self-esteem. [12] Thus, the impact of optimism and pessimism is potentially enormous yet still very unclear.

In this context, even less is known about differences across cultures. While several studies have shown levels of optimism/pessimism to vary across cultures, [1,13-19] findings have been inconsistent in terms of which cultural groups are more or less optimistic. No research to date has compared Asian, African, and Western cultures in the same study.

Recent research among pregnant women has yielded interesting, albeit similarly inconsistent, findings. Lobel et al. [8] found that women who were the least optimistic had babies of the lowest birthweight, even when controlling for gestational age. Moyer et al. [20] found that optimism/pessimism among Ghanaian pregnant women was inversely associated with knowledge of HIV and previous HIV testing. In other words, those who were not tested prior to their pregnancy and had the least knowledge of HIV were the most optimistic. The authors also found that, when compared to a similarly aged sample of non-pregnant women in the United States (U.S.), the Ghanaian women were significantly more optimistic. [20]

The construct of health-related quality of life is one variable that has been linked to optimism/pessimism in past research. [21] In one study, researchers found that, even when health status was controlled, pessimists had significantly worse health-related quality of life (HRQOL) scores than optimists or so-called "realists." [21] In that study, pessimists were those who expected disproportionately negative outcomes associated with their Hepatitis C diagnosis, optimists were those who expected few negative outcomes associated with their Hepatitis C diagnosis, and realists were those who had a fairly accurate perception of the impact Hepatitis C was going to have on their lives. Optimists' HRQOL scores in this study mirrored the scores of the general U.S. population, even though the population being studied (chronic hepatitis C patients) has been shown to have significantly lower QOL than the general population.

The results of the research to date suggest that further examination of the cross-cultural issues in optimism/pessimism and health-related quality of life among pregnant women is warranted. This research was undertaken to explore the differences among a three-sample matched cohort of pregnant women at the same stage in their pregnancies in Ghana, China, and the U.S. The specific aims of this research were to 1) identify if and to what extent optimism and pessimism vary across similar populations of pregnant women in three different countries; 2) determine if and to what extent self-assessed quality of life scores vary among similar populations of pregnant women in three different countries, and 3) determine if optimism and/or pessimism is predictive of or associated with current self-perceived health status and/or self-assessed Health-Related Quality of Life (HRQOL) and how that might vary by culture.

## Methods

### Study Sites

#### China

Data were collected from women presenting for prenatal care at the obstetric outpatient clinic at the Peking University First Hospital between May and July 2006. As one of the largest and most well-known academic medical centers in Beijing, Peking University First Hospital draws both public and private patients from in and around Bejing. Clinics average 600 pregnant women per week and 3000-3500 deliveries per year.

#### Ghana

Data were collected from women presenting for prenatal care at the Obstetrics and Gynecology Clinic at the Noguchi Research Institute/Medical School at the University of Ghana in Accra, Ghana, between May and July 2005. This facility is housed in a public hospital that is also the largest government hospital in Ghana. Patients from all over Ghana travel to this clinic to receive their care. Clinics average 500-600 pregnant women per week and 10,000 - 12,000 deliveries per year.

#### United States

Data were collected from women presenting for prenatal care at the outpatient obstetric clinic at the University of Michigan Health System in Ann Arbor, Michigan, between July 2005 and June 2006. The University of Michigan sees both public and private patients, and patients travel from across Michigan and northern Ohio to seek care. Clinics average 550 pregnant women per week and 3850 deliveries per year.

### Patient population

At all three sites, pregnant women in their last trimester of pregnancy who were 18 years old or older were asked to participate in this research. Women facing an imminent health crisis or those in active labor were excluded. At all three sites, research assistants talked patients through an informed consent form. Translators were used when necessary. Surveys were administered verbally to all participants in Ghana as part of a larger study. [20] In China and in the U.S., surveys were designed to be self-administered, but women were given the option to have the survey administered verbally. (None chose this option.)

### Instruments

The instruments used for the survey in each study location varied slightly, but for the purposes of this analysis, each study site used three key instruments - a demographic questionnaire, the Life Orientation Test (LOT-R), and the Short form 12, or SF-12. The instruments were pilot tested separately in each location, and minor modifications were made to ensure comprehension and comparability across sites. In China and Ghana, the instruments were translated into the dominant language of the region and back-translated into English by native bi-lingual speakers. The original and back-translated versions were compared for consistency, and any inconsistencies were resolved by discussion and consensus among the research team.

A **Demographic and Health Questionnaire **was used to measure patient characteristics that may be associated with optimism, pessimism, HRQOL, or pregnancy outcomes. These include age, number of pregnancies, other medical conditions, previous treatment for mental health problems such as depressed mood or anxiety, previous use of anti-depressants, and self-perceived health status. Women were also asked to rate their perception of the difficulty of their pregnancy on a scale of 1 to 4, with 1 being "extremely easy" and 4 being "extremely difficult."

The **Life Orientation Test - Revised (LOT-R) **[22] is a revision of the original Life Orientation Test [23]. It assesses optimism/pessimism using a series of questions that inquire about an individual's attitudes in daily life. This instrument has been widely validated [24] and used in both Ghana [25] and China.[14] Its 10 items generate an overall score, as well as two possible subscales: affirmation of optimism and affirmation of pessimism. The participant answers each item based on a 5-point scale, with response options ranging from strongly disagree to strongly agree. A higher score relates to a greater level of optimism.

The **Short Form 12, or SF-12**, [26] is a 12-item quality of life instrument derived from the Short Form 36, or SF-36, an instrument used and validated around the world to determine self-assessed health-related quality of life (HRQOL). The SF-36 and shortened SF-12 generate not only summary scales of mental and physical functioning (MCS and PCS), but also a profile of patients' HRQOL across eight domains: physical functioning (PF), role physical (RP), bodily pain (BP), general health (GH), vitality (VT), social functioning (SF), role emotional (RE), and mental health (MH). According to SF-36 developers, 12 of the original SF-36 items accounted for at least 90% of the variance in PCS-36 and MCS-36 in both general and patient populations, and those same 12 items reproduced the profile of the eight SF-36 health concepts sufficiently for studies in which the length of the instrument may be prohibitive.

### Data Collection

All research protocols and survey instruments were reviewed and approved by the institutional review boards at the U.S. and foreign institutions participating.

Pregnant women presenting for prenatal care were approached and asked to participate in a research study. If they expressed an interest, women were asked how far along they were in their pregnancies. Those in their final trimester were taken through a consent form. A research assistant (and translator, when necessary) answered any questions the participant might have and made sure women had a copy of the consent form to keep.

Data were gathered using paper and pencil forms (China, U.S.) and verbal interviews (Ghana). No identifying information was collected from Ghanaian participants, given that collecting post-delivery follow-up data was anticipated to be extraordinarily difficult and thus was not attempted. Hospital registration numbers were collected from Chinese and U.S. patients to allow for post-delivery follow-up for a separate research protocol than that described here. Hospital registration numbers were removed from the original survey and replaced with a unique ID number once the registration number was recorded in a separate location for follow-up purposes.

Responses from the hard copies of the self-administered surveys (China and the U.S.) and the interviewer-administered surveys (Ghana) were entered into an Excel spreadsheet and cleaned.

### Data Analysis

Cleaned data from each site (China, N = 251; Ghana, N = 101; U.S., N = 311) was combined into one large dataset. A data subset was created by matching women on three key variables: maternal age, number of weeks pregnant at the time of enrollment, and number of previous pregnancies. Women were matched within 3 years of age (average age difference across matched sets = 0.85 years); within 1 pregnancy (average difference in number of pregnancies across matched sets = 0.48); and within 5 weeks of gestational age (average difference in gestational age across matched sets = 2.66 weeks). This matching schema was undertaken to attempt to create a sample that was as similar as possible on key variables that could influence optimism/pessimism. Previous research has suggested that age can be related to optimism/pessimism [27], as well as number of previous deliveries [20]. We also postulated that women at different stages of pregnancy might feel differently in terms of optimism/pessimism, and thus wanted to reduce the impact of stage of pregnancy on our results. Given the one child policy in China, "number of previous pregnancies" was used as a key variable rather than number of previous deliveries. This resulted in a matched sample of 168 women, 56 in each country, that were predominantly nulliparous. We refer to these three-way matches as "matched sets" throughout the manuscript.

Frequencies and descriptive statistics were calculated. Means across the three groups were compared using ANOVA with post-hoc pairwise comparisons. In addition, multivariate linear regression analysis was used to create a model to identify the factors most strongly contributing to the overall LOT-R scores. Variables that were significantly associated with the LOT-R in univariate analysis were entered into the multivariate model. The model was reworked using only those variables that maintained significance until the best-fit model was identified. Interactions between key variables were examined as well. A p-value of .05 was taken to be statistically significant.

## Results

### Demographics

Table 1 illustrates the demographic characteristics of women in the sample. Note that two of the three variables upon which women were matched (age and number of previous pregnancies) were not significantly different (see Table 2). In fact, the average difference in age across the matched sets was 0.857 years, with a maximum difference of 3 years. The average difference in number of previous pregnancies was .482, with the highest difference being 2.0, reflected in only 4 of the 56 sets. Women from China were enrolled significantly later in their pregnancies than women in the U.S. (p = .04, mean difference of 1.52 weeks), yet the average difference in gestational age across the matched sets was 2.66 weeks.

**Table 1:** Demographic Characteristics Across the Three-Country Sets

**Variable**	**China (C)** **Mean** **(± SD)**	**Ghana (G)** **Mean** **(± SD)**	**US (U)** **Mean** **(± SD)**	**Significance** **Anova;** **Post-hoc pairwise comparisons when Anova significant**
Age	29.64 (± 4.2)	29.79 (± 3.9 SD)	29.78 (± 4.5)	P = .979 (NS)

Number of weeks pregnant at enrollment	36.22 (± 2.8)	35.99 (± 3.5 SD)	34.7 (± 3.5)	P = .035 CvG: p = .92 (NS) CvU: p = .04 GvU: p = .10 (NS)

Number of times pregnant (including current pregnancy)	2.3 (± 1.2)	2.3 (± 1.1 SD)	2.2 (± 1.2 SD)	P = .877 (NS)

	Percent (N)*	Percent (N)*	Percent (N)*	**Kruskal-Wallis test**

Married	100 (56)	98.2 (54)	76.8 (43)	CvG: p = 1.0 (NS) CvU: p < .001 GvU: p = .10 (NS)

Highest level of education				P < .001
				
- High School graduate or less	30.3 (17)	25.9 (14)	7.2(4)	
- Some College to College Graduate	58.9 (33)	74.1 (40)	42.8 (24)	
- Some Graduate School/Professional School to Completed Graduate Degree	9.0 (5)		44.6 (25)	

Works for money	78.6 (44)	85.7 (48)	64.3 (36)	P = .05

*Ns may not total 56 due to missing data

**Table 2:** Health-Related Variables

**Variable**	**China (C)** **Percent (N)**	**Ghana (G)** **Percent (N)**	**US (U)** **Percent (N)**	**Significance**
**Number of pregnancies**	Mean: 2.3	Mean: 2.3	Mean: 2.2	Anova, p = .877
≤ 1:	30.3 (17)	28.6 (16)	35.7 (20)	
2:	33.9 (19)	32.1 (18)	26.8 (15)	
≥ 3:	35.7 (20)	39.3 (21)	35.7 (20)	

**Number of deliveries**	Mean: 0.11	Mean: 0.98	Mean: 0.76	Anova, p < .001
0:	85.7 (48)	35.7 (20)	50 (28)	CvG: p < .001
1:	10.7 (6)	35.7 (20)	30.4 (17)	CvU: p < .001
2:	0	23.2 (13)	10.7 (6)	GvU: p = .32 (NS)
3+:	0	5.4 (3)	7.2 (4)	

**Description of current pregnancy**	Mean: 1.2 on a scale of 1-4	Mean: 2.0	Mean: 1.9	Anova, p < .001
1 = Very Easy	77.6 (38)	41.1 (23)	29.1 (16)	CvG: p < .001
2 = Somewhat easy	16.3 (8)	28.6 (16)	50.9 (28)	CvU: p < .001GvU: p = .89(NS)
3 = Somewhat difficult	6.1 (3)	4.5 (17.9)	16.4 (9)	
4 = Extremely Difficult	0	12.5 (7)	3.6 (2)	

Seen a healthcare professional for emotional issues*				(Never vs. Ever)
Ever	5.4 (3)	12.7 (7)	38.2 (21)	CvG: p = .54 (NS)
Currently	0 (0)	8.9 (5)	3.7 (2)	CvU: p < .001
Never	94.6 (53)	87.5 (48)	61.8 (34)	GvU: p < .001

Treated for emotional issues with prescription medication*				(Never vs. Ever)
Ever	1.8 (1)	12.5 (7)	18.5 (10)	CvG: p = .15 (NS)
Currently	0 (0)	10.7 (6)	5.5 (3)	CvU: p = .013
Never	98.2 (55)	87.5 (49)	81.5 (44)	GvU: p = .55 (NS)

*Ns may total more than 56. Two separate questions were asked: "Have you ever been treated... yes/no" and "Are you currently being treated...yes/no " Thus the number "currently being treated" is a subset of the number who have "ever been treated." ANOVAs were conducted using the "ever treated" and "never treated" groups for comparison.

All of the women in the Ghanaian sample were married, as were a majority from the China sample (98.2%), and in the U.S. sample, a smaller yet significant percentage of the women (76.8%) were married. Educational variables were assessed slightly differently in Ghana than in China and the U.S., making it difficult to compare across countries. That said, it is clear that the U.S. sample included women with higher levels of education than both China and Ghana (p < .001). Ghanaian women in our sample had the greatest percentage of women working for money (85.7%), China the second greatest (78.6%) and the U.S. the lowest percentage (64.3%) (p = .05).

### Health-related Variables

Table 2 illustrates the health-related variables assessed across the three samples. Note that while the number of pregnancies was not significantly different across the three groups, the number of previous deliveries was significantly different at p < .001.

Women from our sample in China reported having significantly easier pregnancies than women from our Ghanaian and U.S. samples (p < .001), and far more women in the U.S. sample report ever seeing a healthcare professional for help with emotional issues (p < .001).

### Optimism/pessimism

**Figure 1:**
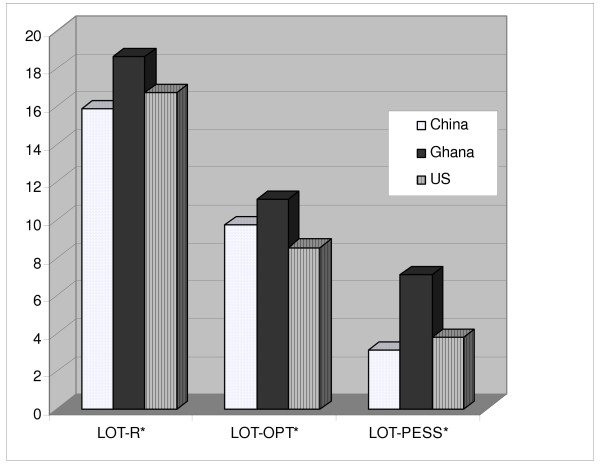
LOT-R Optimism/Pessimism Scores by Country (*ANOVA: p < 0.001). LOT-R = overall optimism; higher score = greater level of optimism LOT-OPT = optimism subscale; higher score = greater level of optimism LOT-PESS = pessimism subscale; higher score = greater level of pessimism

The mean Life Orientation Test Score varied significantly among women in our samples from China, Ghana, and the U.S. (C = 15.85, G = 18.64, U = 16.69; p = .001). Post-hoc pairwise comparisons indicated that the mean scores were significantly different between Ghana and China (p = .001) and China and the U.S. (p = .019), but not between Ghana and the U.S. (p = .49) (See Table 3). Both the LOT-R optimism subscale and the LOT-R pessimism subscale indicate significant differences across country samples as well. (See Figure 1.)

**Table 3:** Optimism/Pessimism Scores by Country

**Variable**	**China (C)** **Mean** **(± SD)**	**Ghana (G)** **Mean** **(± SD)**	**US (U)** **Mean** **(± SD)**	**Significance** **Anova;** **Post-hoc Pairwise comparisons when Anova is significant**
Overall LOT-R	15.85 (± 3.56)	18.64 (± 3.39)	16.69 (± 4.13)	P = .001 CvG: p = .001 CvU: p = .019 (NS) GvU: p = .497 (NS)

LOT-R Optimism Subscale	9.75 (± 2.1)	11.07 (± 1.5)	8.53 (± 2.0)	P < .001 CvG: p = .001 CvU: p = .002 GvU: p < .001

LOT-R Pessimism Subscale	3.11 (± 2.1)	7.09 (± 3.6)	3.76 (± 2.5)	P < .001 CvG: p < .001 CvU: p = .428 (NS) GvU: p, > 001

### Health-Related Quality of Life

**Figure 2:**
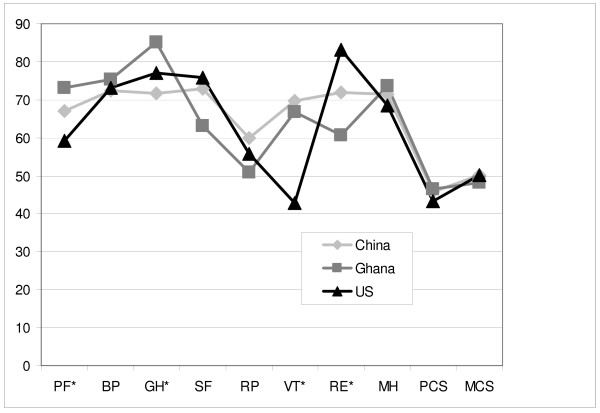
SF-12 Quality of Life Scores by Country (*ANOVA, p < 0.001).

Figure 2 illustrates differences in self-assessed health-related quality of life across the samples. Physical Functioning (PF) was significantly different across the country samples (ANOVA, p = .049), but the difference was primarily between Ghanaian and U.S. samples (post-hoc pairwise comparisons, p = .038). General Health (GH) was significantly different as well (ANOVA, p = .001), and here the difference was primarily between Chinese and Ghanaian samples (p = .001). Vitality (VT) showed significant differences across country samples as well (ANOVA, p < .001), with pronounced differences between Chinese and U.S. samples (p < .001) and Ghanaian and U.S. samples (p < .001), but not Chinese and Ghanaian samples. Finally, Role Emotional (RE) scores were also significantly different across country samples (ANOVA, p < .001), but the difference was most pronounced between Ghanaian and U.S. samples (p < .001).

### Correlates of Optimism/pessimism

Among all the women across the three samples, univariate analysis indicated that overall LOT-R optimism/pessimism scores were significantly associated with country of origin (p = .001; Ghanaian women sampled had the highest scores, U.S. women sampled had scores in the middle, Chinese women sampled had the lowest scores), educational attainment (p = .046; optimism increased with education level), whether women worked for money (p = .006; working women were more optimistic than non-working women), and number of previous deliveries (p = .004; those with the fewest and the most deliveries were the most optimistic - those with 2 previous deliveries were the least optimistic).

Women who had never seen nor were currently seeing a health care provider for emotional issues were more optimistic than those who had ever seen or were currently seeing a health care provider for emotional issues. (p = .002, p = .001 respectively) Treatment for mental health issues with medication was another variable that was associated with LOT-R scores: those ever treated and those currently being treated with medication were significantly less optimistic than those who had never been treated. (p < .001, p = .005 respectively)

Women's self-reported experience of the ease or difficulty of the current pregnancy was also associated with LOT-R scores (p = .007). Those who reported having the easiest pregnancies had the highest levels of optimism.

LOT-R scores were positively correlated with SF-12 mental health summary scores (p = .001), vitality subscale scores (p = .041), and mental health subscale scores (p < .001). The LOT-R optimism subscale was significantly associated with the same scales (MCS, p = .018; VT, p < .001; MH, p = .001), as well as the general health subscale (GH, p = .002). The LOT-R pessimism subscale was associated with the mental health summary score (p = .013), the role physical subscale (p = .032), the role emotional subscale (p = .034), and the mental health subscale (p = .038).

Note that LOT-R scores were not associated with the self-reported presence of any ongoing health issues.

Multivariate linear regression analysis indicated four key variables that predicted approximately 20% of the variance (adjusted R square = .199) in overall LOT-R scores: country of origin (p = .015), working for money (p = .025), level of education (p = .001), and ever being treated for emotional issues with medication (p = .002). No significant interactions were identified.

## Discussion

These data suggest that in a three-country cohort of pregnant women matched on age, number of weeks pregnant, and number of previous pregnancies, significant differences existed with regard to both optimism/pessimism and health-related quality of life. According to the LOT-R, Ghanaian pregnant women in our sample were the most optimistic and Chinese women in our sample were the least optimistic of the matched sets. Interestingly, Ghanaian women sampled also reported higher levels of pessimism than Chinese and U.S. women sampled. This is consistent with research that suggests optimism and pessimism may not be mutually exclusive constructs.[28] Physical functioning, general health, vitality, and role emotional subscales of the SF-12 also indicate significant differences by country per our samples - U.S. women sampled had the lowest physical functioning and vitality scores, Ghanaian women sampled had the highest physical functioning and general health scores, and Chinese women sampled had the lowest general health scores and highest vitality scores.

Correlates of optimism/pessimism included country of origin, educational attainment, working for money, number of previous deliveries, and having ever seen a healthcare provider for emotional issues. Self-reported ease or difficulty of the pregnancy was also associated with LOT-R scores.

Perhaps most striking in these findings is that we were able to create a multivariate linear regression model that predicted approximately 20% of the variance in overall LOT-R scores. The key variables were country of origin, working for money, level of education, and ever being treated for emotional issues with medication. What is particularly fascinating about this model is that country of origin somehow remains a strong factor in the model - even with variables that might be seen as proxies for country of origin (education, working for money, ever being treated for emotional issues). It raises a question about the potential differences that underlie being Ghanaian, Chinese, or American that yield differential LOT-R scores. It also raises questions about what accounts for the remaining 80% of the variance. Further research with more comprehensive instrumentation is necessary to begin to answer these questions.

There are several limitations to this research. First, the study design does not allow for the definitive determination of a causal relationship - we can only report observed associations. Second, in each setting, the women recruited represented a convenience sample from one hospital in one city, minimizing the ability to generalize based upon our findings. Third, we created our matched sets based on three variables we believed were most important to control for at the outset: maternal age, gestational age, and number of previous pregnancies. Given the one child policy in China, we did not match on number of previous deliveries - as that would have eliminated all but the nulliparous women in Ghana and the U.S. and reduced our sample size to an untenably low level. However, upon analysis it was discovered that the number of previous deliveries was significantly different across countries. In China, women were more likely to have had a previous pregnancy that did not result in a delivery. Whether these were induced or spontaneous terminations is not known, although previous research indicates that more than half of women in China have had at least one abortion. [29]

It is possible that Chinese women who have had the same number of pregnancies as their African and North American counterparts but have not had as many deliveries are in some way inherently different. Our univariate analysis did indicate that number of deliveries was associated with overall LOT-R scores (p = .004), but this significance was not sustainable in the multivariate logistic regression model.

Note as well that the samples differ significantly on a few key variables. First is in terms of educational attainment. In Ghana and China, approximately a third or respondents were high school graduates or less, compared to only seven percent in the U.S. Our data suggest that educational attainment is associated with optimism/pessimism and health-related quality of life. What is less clear is through what mechanism. Further research is needed to disentangle these associations. The second key difference in the samples is related to their reports of current and previous treatment for emotional issues. While nearly 40% of U.S. women in our sample reported ever seeking care for emotional issues, only 5% of Chinese women in our sample said the same. While this may reflect true differences in need for mental health care, more likely it reflects social norms and access issues surrounding treatment for mental health issues. Nonetheless, such differences are indicative of the likely many other variables that would differ across cultures that were not measured in this study and that might have an effect on optimism/pessimism or self-assessed health-related quality of life. Finally, the samples were likely to be very different in terms of standard of living. Women in Ghana were recruited from a public hospital, suggesting lower income participants, whereas women in China and the U.S. were either 'public' or 'private,' depending upon the type of insurance being used. We attempted to assess income to sort out these differences in a meaningful way, but there were numerous challenges. The first is the distinction between family and individual income. In China, family income may or may not include extended family. There may be four - or even six - adults in one household, awaiting the arrival of a single child. In Africa, family income was challenging for some respondents to answer, whether due to lack of knowledge or the challenge of translating non-currency income into survey response options. In addition, assuming for a moment that our survey instrument accurately collected and recorded a representative gradient of incomes in each culture, how does one compare being 'low income' in the U.S. versus being 'low income' in sub-Saharan Africa? The combination of missing data and uncertain interpretation of our income-related items made meaningful comparisons challenging at best, misleading at worst, thus income data were removed from analysis.

Another limitation to this research is that we were unable to suitably control for or verify "difficult" pregnancies. We did not have independent corroboration of women's reported symptoms or reported ratings of the ease or difficulty of their pregnancies. Our sole measure of the ease or difficulty of women's pregnancies was their self-report. That said, the LOT-R measures what is referred to as dispositional (trait) optimism/pessimism - or something that is seen as relatively stable over time. Other measures of situational (state) optimism may have been more responsive to changes over time and may have been influenced by easy or difficult pregnancies. However, for the purposes of this research, we believe that a measure of dispositional optimism/pessimism is the most appropriate and that it should not have been differentially influenced by women's pregnancies. This is corroborated by the finding that despite reporting the easiest pregnancies of the three groups of women, Chinese women in our sample reported the lowest levels of optimism.

Another limitation to this research is the uncertainty surrounding the comparability of the constructs of optimism/pessimism across cultures. In a review of Chinese folk wisdom of behavioral health, researchers cite that for Chinese subjects, being optimistic means to be able to accept one's current life conditions positively rather than to expect good things to occur in one's life.[30] This is a more present-focused interpretation of optimism, rather than the future-focused interpretation most commonly used in the West. In the West, optimism is defined as the expectation that good things will happen to you in the future. Given these potentially contradictory definitions, future research needs to identify and clarify definitions of optimism and pessimism as commonly understood in different areas of the world.

Similarly, it is unclear how Ghanaians would articulate the concept of optimism. Research in Africa has shown that optimism has been inversely correlated to income and a number of other indicators of higher standards of living and positively correlated with a number of variables associated with deep poverty. [31] This finding - if real - raises several critical questions. Are those who are optimistic in the midst of poverty the ones that survive? Is optimism a result of the perception that if one is extremely poor it can't get much worse, so it must be getting better? Are those with higher incomes and better standards of living so afraid of losing it all that they steel themselves against that potential by assuming the worst? Clearly more research is needed to begin to tease out some of these issues.

One final limitation is the inconsistency of data collection methodology across sites. Data were colleted verbally in Ghana, whereas data were collected via self-reported surveys in China and the U.S. We do not believe this difference significantly impacted the outcome variables in question, however it is possible there may be differences attributable to the method of data collection.

## Conclusion

In conclusion, these results raise some critical questions worthy of further exploration. Rigorous studies of cross-cultural differences are not only methodologically, but also logistically, very challenging. That said, research needs to address the question of whether the underlying constructs of optimism/pessimism and health related quality of life are conceptualized similarly across cultures. In addition, further research is warranted that explores the potential link between psychosocial constructs such as these and measurable health outcomes. Only then can researchers and practitioners explore the possibility of interventions to influence what have typically been seen as stable constructs.

## Competing interests

The authors declare that they have no competing interests.

## Authors' contributions

CAM, HY, YK, and FA conceived of the study, supervised the collection and analysis of data, participated in manuscript drafting and review, and gave final approval for the version to be published. HY supervised data collection and provided guidance for analysis in China. YK, RA, and FA supervised data collection and provided guidance for analysis in Ghana. AG, MB and MR collected and analyzed data from the USA and participated in manuscript drafting and revision. YZ, YM, and YE participated in data collection and analysis in China, as well as participated in manuscript drafting and revision. IK, JG, CC, and GE worked together to collect and analyze data in Ghana, as well as providing critical input to the methodology section of the manuscript pursuant to issues in Ghana. All authors contributed to the manuscript development, revision, and final approval.

## Pre-publication history

The pre-publication history for this paper can be accessed here:


